# Antifungal Agents in Wood Protection—A Review

**DOI:** 10.3390/molecules27196392

**Published:** 2022-09-27

**Authors:** Magdalena Woźniak

**Affiliations:** Department of Chemistry, Faculty of Forestry and Wood Technology, Poznan University of Life Sciences, Wojska Polskiego 75, 60625 Poznań, Poland; magdalena.wozniak@up.poznan.pl

**Keywords:** antifungal agents, wood protection, natural compounds, plant extracts, phenolic compounds

## Abstract

The biodegradation of wood and wood products caused by fungi is recognized as one of the most significant problems worldwide. To extend the service life of wood products, wood is treated with preservatives, often with inorganic compounds or synthetic pesticides that have a negative impact on the environment. Therefore, the development of new, environmentally friendly wood preservatives is being carried out in research centers around the world. The search for natural, plant, or animal derivatives as well as obtaining synthetic compounds that will be safe for humans and do not pollute the environment, while at the same time present biological activity is crucial in terms of environmental protection. The review paper presents information in the literature on the substances and chemical compounds of natural origin (plant and animal derivatives) and synthetic compounds with a low environmental impact, showing antifungal properties, used in research on the ecological protection of wood. The review includes literature reports on the potential application of various antifungal agents including plant extracts, alkaloids, essential oils and their components, propolis extract, chitosan, ionic liquids, silicon compounds, and nanoparticles as well as their combinations.

## 1. Introduction

Wood, as a renewable and natural organic material, plays an important role in various branches of industry, mainly in the construction and furniture fields. However, one of the most serious problems limiting the use of wood and wood products, especially in outdoor applications, is their susceptibility to degradation caused by many organisms including fungi, bacteria, or termites [1,2]. 

**Figure 1:**
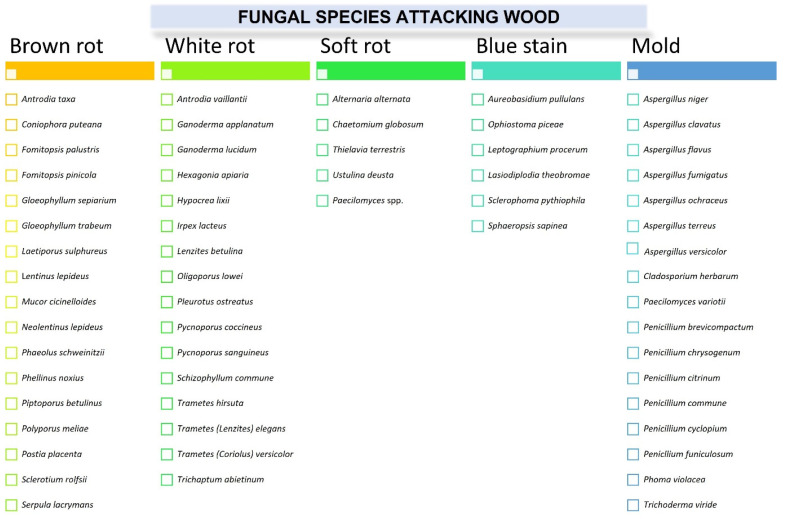
The species of fungi that can attack wood.

Wood decay fungi can be divided into three main groups when considering their capability for the biomineralization of wood’s main structural components such as cellulose, lignin, and hemicelluloses: white rot, brown rot, and soft rot [3,4]. Wood can also be attacked by less destructive molds and blue stain fungi, which affect the aesthetic value of wood and wood products [1,4]. Brown rot fungi, belonging to Basidiomycetes can degrade wood polysaccharides and only partially modify lignin, resulting in brown material consisting of oxidized lignin [5,6]. Representative examples of brown rot fungi include *Coniophora puteana*, *Gloeophyllum trabeum*, *Laetiporus sulphureus*, *Piptoporus betulinus*, *Postia placenta*, and *Serpula lacrymans* [5,6,7]. White rot fungi can depolymerize all three major wood components, while lignin degradation by this class of fungi results in a whitish color and a fibrous texture of decayed wood [1,4]. Most white rot fungi belong to the “simultaneous” white fungi including *Trametes versicolor*, *Heterobasidion annosus*, *Xylaria polymorpha*, *Irpex lacteus*, and *Daldinia concentrica*, which simultaneously degrade all major components of the wood cell wall. In turn, “selective” white rot fungi such as *Ganoderma australe*, *Phlebia tremellosa*, *Ceriporiopsis subvermispora*, and *Phellinus pini* delignify wood by preferentially digesting lignin and hemicelluloses, leaving cellulose relatively undegraded [1,4,6]. Most soft rot fungi belong to Ascomycota species and can attack wood in two forms. In type I, characteristic cavities are produced in the cell walls by fungi, while in type II, hyphae located in the cell lumina cause cell wall erosion [1,6]. Soft rot fungi include both species from Ascomycetes (*Chaetomium globosum*, *Ustulina deusta*, etc.) and Deuteromycetes (*Alternaria alternata*, *Thielavia terrestris*, *Paecilomyces* spp., etc.) [6,7]. In turn, molds and blue strain fungi (sapstain) degrade the nutrient reserves of the wood (mainly wood extractives and water-soluble components) and usually do not cause significant damage to the wood structure. Blue strain fungi include *Ophiostoma piceae* and *Lasiodiplodia theobromae*, while molds that can attack wood and wood products include, for example, *Aspergillus niger*, *A. versicolor*, *Penicillium brevicompactum*, and *Rhizopus* spp. [6,7]. Examples of fungal species that can attack wood and are discussed in this paper are presented in Figure 1. 

In order to extend the service life of wood and wood products, various methods and techniques are applied including chemical processing (furfurylation or acetylation), thermal modification, or impregnation with numerous substances and chemicals [8,9,10,11,12]. Wood preservatives are fungistatic or fungitoxic chemicals including oil-borne preservatives such as pentachlorophenol or creosote, which are still used for industrial applications where the wood will be in limited contact with humans. Wood is also impregnated with waterborne wood preservatives, which nearly all contain copper as a biocide [10,13]. However, most traditional wood preservatives, due to their toxicity, cause serious environmental hazards. An example of this preservative can be a copper chromate arsenate (CCA) water-borne solution, which is a restricted chemical product in most countries due to the potential environmental and health risks from skin contact with CCA residues from impregnated wood [14]. Therefore, the development of new wood preservatives with a low environmental impact is being carried out in research centers around the world. Numerous natural substances, mainly of plant origin including essential oils and their components, plant extracts, phenolic compounds, or alkaloids have been investigated as potential antifungal agents in wood protection [15,16,17,18,19]. However, substances of animal origin such as beeswax, chitosan, or snail peptides have also been examined as antifungal substances improving the wood durability [15,19,20,21]. There are also various synthetic compounds with antifungal activity that are being studied for use in wood protection including but not limited to ionic liquids, silicon compounds, or nanoparticles [7,22,23]. One of the most important drawbacks of using natural substances in wood protection, especially wood used outdoors, is their susceptibility to leaching. Therefore, various methods or techniques have been used to prevent the leaching of natural substances from the wood structure including the application of formulations consisting of natural substances with a combination of synthetic compounds—cyclodextrins or silicon compounds [24,25,26]. Numerous literature reports have indicated that there are many substances and chemical compounds that show a fungistatic effect and are characterized by a much lower negative impact on the natural environment than the commonly used preservatives applied in the protection of wood.

The aim of this review was to prove the information in the recent literature data describing antifungal agents for application in wood protection. The article presents the literature data on the substances and chemical compounds that are not considered as traditional fungicides used in wood protection. The work focuses both on natural substances of plant and animal origin as well as on the synthetic compounds that can be used in ecological wood protection. The potential use of preparations containing a mixture of various antifungal agents is also described in the paper. The review presents both the results of the antifungal activity of the agents against microorganisms attacking wood and the results of the antifungal resistance of wood treated with these agents. 

The articles including original and review articles that were selected for pre-review based on electronic and manual searches using the defined search criteria. The articles were searched on the basis of the defined keywords (including ‘wood’, ‘wood protection’, ‘natural products’, ‘essential oils’, ‘antifungal activity’, ‘phenolic compounds’, ‘alkaloids’, ‘flavonoids’, ‘terpenes’, ‘polyphenols’, ‘phenols’, ‘silicon compounds’, ‘natural preservatives’, ‘antifungal agents’, ‘herbal drugs’, ‘plant extracts’, ‘monoterpenes’, ‘microbial metabolites’, ‘chitosan’, ‘synthetic derivatives of natural products’, ‘animal extract’) in the Web of Science, Scopus, Science Direct, and PubMed databases. The obtained records were included and excluded on the basis of the following criteria. Data inclusion criteria included: (a) articles involving extracts of plant and animal sources as well as their fractions and ingredients, tested for activity against fungi attacking wood; (b) studies related to derivatives of natural products obtained by synthesis analyzed against antifungal effects; and (c) synthetic compounds with low environmental impact tested against antifungal action. Exclusion criteria included: (a) data duplication and titles or contents that do not meet the inclusion criteria; (b) literature reports on the antifungal activity of natural products or their derivatives as well as synthetic compounds against fungi that do not attack wood or tree; and (c) research with the use of synthetic, traditional chemicals applied in wood protection.

## 2. Plant-Derived Antifungal Agents

Plants are a source of many compounds that exhibit broad biological activity including action on various species of fungi. An important group of plant constituents includes secondary metabolites, which can be divided into four classes: phenolic compounds, alkaloids, terpenoids, and sulfur-containing compounds [27]. These phytochemicals play many roles including protecting plants from pathogens and environmental stress as well as mediating interactions between organisms [27,28]. Additionally, plant derivatives such as essential oils or extracts obtained from various plant materials exhibit a biological effect. Therefore, chemical compounds and substances of plant origin may be of interest in the development of new and environmentally friendly wood preservatives. In addition, an important advantage of plant-derived products is their rich variety and high availability as well as the fact that bioactive compounds can be obtained from plant waste. The literature indicates that many different substances of plant origin have been tested in the protection of wood including, for example, essential oils, tannins, terpenes, plant extracts, phenolic compounds, alkaloids, propolis extract, plant oils, or resins [15,17,18,19,29,30,31]. This section discusses the antifungal activity of the selected groups of plant derivatives including essential oils and their ingredients, alkaloids, extracts from various plant species, and propolis extract. 

### 2.1. Alkaloids

Plant alkaloids are one of the largest groups of natural products, which possess multiple biological effects including antimicrobial, anti-inflammatory, mutagenic, and carcinogenic activity [32,33,34]. According to the literature reports, canthin-6-one and its derivatives are one of the most widely studied alkaloids with antifungal activity. Cathin-6-one and 5-methyloxycathin-6-one showed higher activity against *A. fumigatus*, with a minimal inhibition concentration (MIC) equal to 14.2 and 50.0 µM/L, respectively, compared to ketoconazole (MIC = 94.2 µM/L) [35]. In turn, cathin-6-one isolated from *Simaba ferruginea* A. St.-Hill exhibited good activity against *A. niger* (MIC = 6.25 µg/mL) and *A. fumigatus* (MIC = 3.125 µg/mL), when compared with the reference compound—chloramphenicol (MIC = 3.125 µg/mL) [36]. Pyrrolizidine alkaloids (subulacine N-oxide, 7-angeloyl heliotrine, retronecine, heliotric acid, heliotrine) isolated from *Heliotropium subulatum* presented activity against *A. niger*, *A. fumigatus*, and *P. chrysogenum* [37]. In turn, Kwaśniewska-Sip et al. [38] examined 74 quinolizidine and bisquinolizidine alkaloids and found that only 11 compounds—cytisine and its derivatives, mainly halogenated (e.g., N-boccytisine, m-bromo-benzylcytisine, p-chloro-benzylcytisine, m-jodo-benzylcytisine and spirocytisine)—showed activity against *A. niger*. 

Caffeine is an alkaloid that has recently been extensively researched for its use in wood preservation. Literature reports have indicated that wood treated with a caffeine solution showed resistance to fungal attack, however, the effectiveness of caffeine as a natural fungicide depends on its concentration, the type of impregnated wood, and the fungus strain used [39,40,41]. Various wood species impregnated with an aqueous solution of caffeine were resistant against white rot fungus (*T. versicolor*), brown rot fungi (*C. puteana*, *P. placenta* and *G. trabeum*), soft rot fungus (*Ch. globosum*), wood-staining fungi (*A. pullulans* and *S. pythiophila*), and molds (*A. niger*, *A. terreus*, *C. herbarum*, *P. variotii*, *P. cyclopium*, *P. funiculosum*, *P. brevicompactum*, *P. violacea*, and *T. viride*) [39,40,41,42,43,44,45]. Kobetičová et al. [46] assessed the effect of non-toxic methylxanthines including caffeine and its metabolites—theobromine and theophylline on the growth of four wood-destroying fungal species (*S. lacrymans*, *C. puteana*, *G. sepiarium*, and *T. versicolor*) in the agar test. The results indicated that caffeine exhibited a 100% inhibitory effect on all fungal strains, in contrast to theobromine, which was not effective in this regard. In turn, theophylline showed a variable effect on the tested fungi—the most sensitive species was *T. versicolor*, followed by *S. lacrymans*, *G. sepiarium*, and the least sensitivity was related to *C. puteana* [46]. Moreover, the research into the chemical interaction between caffeine and wood components has indicated that caffeine was able to mainly bond with lignin [47,48]. The advantages of the application of caffeine as a wood preservative is its effectiveness in protecting wood against fungi, even in a relatively low concentration (1–2%) and the possibility of obtaining it from the waste of the coffee industry. However, as reported in the literature, caffeine is susceptible to leaching from the structure of the treated wood, which makes it impossible to use wood impregnated with caffeine in outdoor conditions [39,41]. 

### 2.2. Essential Oils and Their Components

**Figure 2:**
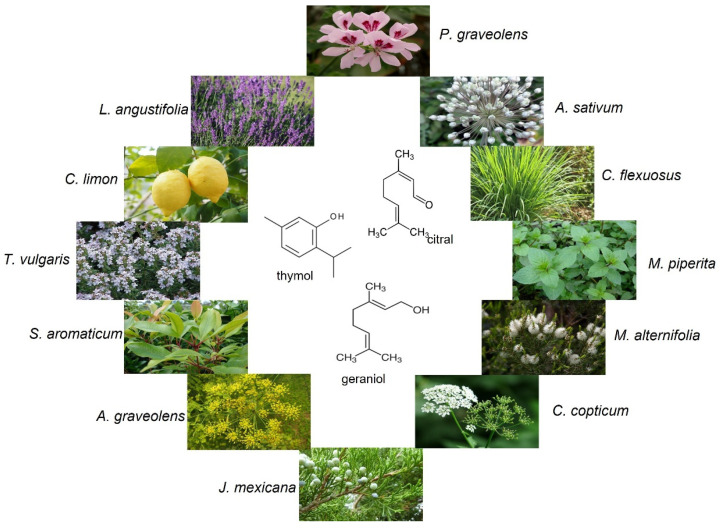
The plant sources used to obtain essential oils and their constituents that were tested as an ecological preservative in wood protection.

Worldwide interest in essential oils and their ingredients in the application of ecological wood protection research is growing, and the number of new essential oils with biological activity is constantly increasing. Therefore, essential oils and their components constitute an important and numerous group of ingredients of preparations with potential use in ecological wood protection. Examples of plant sources used to obtain essential oils that were tested as an ecological preservative in wood protection are shown in Figure 2.

In the literature, the activity of essential oils and their constituents against both wood decay fungi (*C. puteana*, *C. versicolor*, *P. placenta*, *G. trabeum*, *L. betulina*, *L. sulphureus*, *P. coccineus*, *T. abietinum*, *O. lowei*, *A. taxa*, *F. pinicola,* and *P. schweinitzii*) as well as mold fungi (*A. niger*, *Fusarium subglutinans*, *T. viride*, *P. brevicompactum*, and *P. chrysogenum*) have been investigated [16,49,50,51,52,53,54,55]. The essential oils used in the antifungal studies were obtained from numerous plants including plant species growing in various parts of the world as well as species characteristic for a given climate and geographical zone. The essential oils of lavender, clove, oregano, thyme, and sweet flag are among the most frequently studied oils as potential preservatives in wood protection [16,30,56]. 

Xie et al. [57] examined the activity of six essential oils—*Origanum vulgare*, *Cymbopogon citratus*, *Thymus vulgaris*, *Pelargonium graveolens*, *Cinnamomum zeylanicum* and *Eugenia caryophyllata*—against white rot (*T. hirsuta*) and brown rot (*L*. *sulphureus*) fungi, which indicated that *O. vulgare* was the most toxic to the tested wood rot fungi. The activity of essential oils from the leaves of four eucalyptus species, namely *Eucalyptus urophylla*, *E. grandis*, *E. camaldulensis* and *E. citriodora* against fungal species (*A. niger*, *A. clavatus*, *Ch. globosum*, *Myrothecium verrucaria*, *P. citrinum*, and *T. viride*) were evaluated by Su et al. [58]. The authors demonstrated that the essential oil from *E. citriodora* was extensively effective against all of the tested fungal species [58]. The essential oils from the *E. camaldulensis* aerial parts, *Citrus aurantium* leaves, and *Citrus sinensis* peels showed antifungal activity, while the essential oil from *C. sinensis* peels showed the highest activity among the tested oils against the examined fungi: *A. niger* (MIC 6 µL/mL), *A. flavus* (MIC 12 µL/mL), and *A. terreus* (6 µL/mL) [59]. The results described by de Medeiros et al. [60] indicated that the essential oil from native Brazilian savannah shrub—*Lippia origanoides* showed a stronger growth inhibition of *G. trabeum* and *T. versicolor* than a commercial fungicide. The effect of the essential oils of eighteen Egyptian plants against wood decay fungi—*H. apiaria* and *G. lucidum*—was assessed by Mohareb et al. [61]. The six oils (*C. sempervirens, C. limon, T. occidentalis, S. molle*, *A. monosperma*, and *P. graveolens*) caused a significant reduction in the Scots pine wood mass loss after 6 weeks of fungal exposure. The essential oils from *A. monosperma* showed the strongest inhibitory effect against *G. lucidum*, and the main components of the oil were β-thujone, chrysanthenone, and α-thujone. In turn, the oil of *C. limon* revealed the highest reduction in wood mass loss caused by *H. apiaria*, and the main ingredients of this oil were limonene, β-pinene, and γ-terpinene [61]. The results of research on the resistance of rubberwood treated with essential oils (peppermint and eucalyptus) and their components (menthol and eucalyptol) against mold fungi (*A. niger* and *P. chrysogenum*) and wood decay fungus (*T. versicolor*) indicated that peppermint oil and its main component menthol showed a higher antifungal activity than eucalyptus oil and its main ingredient, eucalyptol [62]. In turn, Bahmani and Schmidt [30] examined the activity of Oriental beech (*Fagus orientalis*) and Loblolly pine *(Pinus taeda)* wood treated with sixteen essential oils against molds (*A. niger* and *P. commune*), brown rot fungus (*C. puteana*), white rot fungus (*T. versicolor*), and soft rot fungus (*Ch. globosum*) and found that thyme, lavender, and lemon grass essential oils exhibited the most effective action against all of the tested fungi species. Literature reports indicate that essential oils have potential as agents that protect wood against the destructive action of some fungal species. However, the application of some essential oils for wood treatment also has some disadvantages. Panek et al. [16] found that the color of beech wood treated with various essential oils changed individually in accordance with the natural color of the applied oils. Birch, sweet flag, tea tree, savory, and oregano oils, which are characterized by a yellow tone, caused more yellow tones of the treated wood [16]. Moreover, essential oils are susceptible to being leached from the structure of treated wood, which cause higher weight losses of treated wood exposed to fungal action [16,56]. A summary of the research on the antifungal activity of essential oils as potential preservatives in wood protection is presented in Table 1.

The literature data also describe the effect of the individual components of essential oils on various strains of fungi. The studies on the activity of forty-one monoterpenes against three white rot fungi (*T. hirsuta, S. commune*, and *P. sanguineus*) showed that eugenol, thymol, carvacrol, and citral showed the highest fungicidal properties among all of the tested compounds [17]. Xie et al. [57] found that the six examined components of essential oils (carvacrol, citral, thymol, citronellol, cinnamaldehyde, and eugenol) exhibited strong activity against the wood decay fungi *T. hirsuta* and *L. sulphureus*, and carvacrol had the best fungicidal activity against the tested fungal strains. In turn, Abbaszadeh et al. [67] examined the activity of four constituents of essential oils—thymol, carvacrol, menthol and eugenol—against various fungal strains such as *A. niger, A. fumigatus, A flavus, A. alternata, P. citrinum, P. chrysogenum,* and *Fusarium oxysporum* and indicated that all compounds were effective to varying degrees against the tested fungi, however, carvacrol displayed the highest efficacy. The study of the activity of eugenol and its congeners against three white rot fungi (*T. hirsuta, S. commune,* and *P. sanguineus*) indicated that eugenol showed a stronger antifungal effect that its derivatives [68]. Voda et al. [69] determined the activity of twenty-two essential oil phenols, phenolic esters, and aromatic aldehydes against wood-decaying fungi—*T. versicolor* and *C. puteana*, and the research showed that the tested compounds exhibited various antifungal activity. The activity of the essential oil ingredients was related to their chemical structure—compounds with alkyl or alkenyl groups in aromatic rings characterized by a higher antifungal effect than the compounds that contained oxygenated substituents in aromatic rings [69]. The research by Zhang et al. [70] indicated that citral had an inhibitory effect on common bamboo molds (*A. niger, T. viride* and *P. citrinum*), and its concentration of 100 mg/mL showed an inhibitory rate of over 100% against the examined fungi. In turn, bamboo wood treated with citral in a concentration twice as high (200 mg/mL) was characterized by the lack of molds growing on the wood surface [70]. A summary of the research on the antifungal activity of essential oil constituents as potential preservatives in wood protection is presented in Table 2.

### 2.3. Propolis Extract

Propolis is a plant-derived product collected by bees from the flowers, buds, and exudates of various trees and plants, and modified with bee secretions and wax [77,78]. It is characterized by multiple biological effects including antimicrobial, antioxidant, anticancer, or anti-inflammatory activity [77,79,80,81,82]. These biological properties of bee glue are related to its chemical composition and, more specifically to phenols [83]. In turn, the chemical composition of propolis is mainly related to the local flora in the place where the apiary is located as well as to the time and technique of its collection, or the species of bees [77,83,84,85,86]. Thanks to the antimicrobial activity of the extracts, propolis has been used in the research on ecological wood protection.

Literature reports have indicated that extracts of propolis from various geographic origins inhibited the growth of fungal strains such as *A. niger*, *A. flavus*, *A. fumigatus*, *A. versicolor*, *P. chrysogenum*, *P. variotii*, *T. viride*, *T. versicolor*, and *Ch. globosum* [87,88,89,90,91,92]. Ethanolic extract from Argentine propolis showed an inhibitory effect on the hyphal fungal growth of *G. applanatum*, *T. elegans*, and *S. commune* as well as *P. sanguineus*, which were isolated from local decaying wood [93]. Scots pine wood treated with 7% methanolic extract of Turkish propolis showed resistance to brown rot fungus—*N. lepideus* (mass loss of 4.2%) and white rot fungus—*T. versicolor* (mass loss of 2.5%) compared to the untreated wood samples [94]. In turn, paulownia wood impregnated with Turkish propolis extract at 7% concentration was characterized by lower resistance to brown and white fungi, with a mass loss of 11.6 and 12.3%, respectively, compared to propolis-treated pine wood [94]. Norway spruce treated with the Slovenian ethanolic propolis extract exhibited quite high resistance to *T. versicolor* (mass loss of 4.60%), *G. trabeum* (mass loss of 7.20%), and *A. vaillantii* (mass loss of 5.28%) compared to the untreated wood [95]. The results of studies on poplar wood treated with Spanish propolis extract at a concentration of 5 to 40 mg/mL and exposed to *T. versicolor* for 4, 8, 12, and 16 weeks showed that the protective effect of the propolis extract depended on the propolis concentration and time of wood exposure to fungi. However, wood impregnated with propolis extract in all of tested concentrations exhibited a higher resistance against fungus than the untreated wood samples [96]. Scots pine wood impregnated with an ethanolic extract of propolis collected from Poland showed resistance to *C. puteana* and the antifungal protection of the propolis extract was related to its concentration. An increase in the concentration of the propolis extract used for wood impregnation from 3 to 30% resulted in a decrease in the value of wood weight loss from 31.6% to 2.7%. However, pine wood impregnated with 12% propolis extract already showed good resistance to *C. puteana*, with a weight loss of 3.3% compared to a weight loss of the control samples of 48.8% [97]. 

The biological activity of poplar-type propolis is caused mainly by the presence of phenolic compounds including flavonoids and aromatic acids [81,98]. In turn, the pharmacological effect of other types of propolis (e.g., from the tropical zone) is related to the presence of various bioactive compounds including lignans, coumarins, and stilbenes [82,91]. Therefore, the antifungal activity of propolis extracts may vary depending on its origin and other factors such as the fungal strain or solvent used in the extraction process or time and method of harvesting [84,99,100]. 

### 2.4. Plant Extracts and Other Plant Derivatives

Plant extracts are another group of plant derivatives that have been intensively developed as natural wood preservatives. The extracts are obtained from numerous plant species and from various plant parts including bark, leaves, fruits, flowers, buds, seeds, or wood [18,19,101,102]. Numerous plant extracts with biological activity have been used in the research on their possible application in environmentally friendly wood protection.

The antifungal activity of plant extracts and their ingredients have been examined by in vitro methods using the disk-diffusion and broth or agar dilution methods as well as determining the resistance of wood impregnated with the plant extracts to the action of microorganisms. Kawamura et al. [103] examined the antifungal activity of 35 extracts from 15 species of Malaysian wood and found that the methanolic extract from *Neobalanocarpus heimii* bark and *Endospermum malaccense* inner wood showed the highest activity against white rot fungus *P. sanguineus*, while methanolic extracts from *N. heimii* bark and *Cinnamomum porrectum* heartwood showed moderate activity against the brown rot fungus *G. trabeum*. In turn, Özgenc et al. [29] examined the in vitro antifungal effect of tree bark extracts from maritime (*Pinus pinaster* L.), iron (*Casuarina equisetifolia* L.), mimosa (*Acacia mollissima* L.), Calabrian pine (*Pinus brutia* Ten.), and fir (*Abies nordmanniana*). The results indicate that the maritime and fir tree bark extracts exhibited good resistance to *T. versicolor*, while iron and mimosa tree bark extracts were more resistant to *C. puteana* [29]. The antifungal activity of 17 hot water extracts from vegetable origin household waste against the wood decay fungi *P. placenta*, *T. versicolor*, and *G. trabeum* in vitro was determined by Barbero-Lopez [104]. The 14 extracts showed activity against the tested fungi, although their efficacy varied significantly, depending on the studied fungal species and the type of extracts. The banana peel extract showed the best antifungal activity, while onion, tangerine, and watermelon peel extracts caused a significant inhibition of some of the tested fungi [104]. The antifungal activity of plant extracts against fungi species attacking wood are presented in Table 3. 

Tascioglu et al. [112] examined the antifungal activity of the Scot pine (*P. sylvestris* L.), beech (*F. orientalis* L.), and poplar (*P. tremula*) wood specimens treated with various concentrations of three commercial plant extracts of mimosa (*A. mollissima*), quebracho (*Schinopsis lorentzii*), and pine (*P. brutia*) bark against white rot (*T. versicolor* and *P. ostreatus*) and brown rot fungi (*F. palustris* and *G. trabeum*). The results indicated that wood impregnated with 9% and 12% mimosa and quebracho extracts exhibited resistance against all types of tested fungi. In turn, pine bark extract was ineffective against the examined fungal species, even when it was used in wood treatment at the highest concentration of 12% [112]. Salem et al. [113] investigated the antifungal effect of three natural extracts applied on three wood species (*P. sylvestris*, *P. rigida*, and *F. sylvatica*) against five fungi—*A. alternata*, *F. subglutinans*, *Ch. globosum*, *A. niger* and *T. viride.* Among the tested plant extracts (*Pinus rigida* (heartwood), *Eucalyptus camaldulensis* (leaves), and *Costus speciosus* (rhizomes)), the extracts from *P. rigida* heartwood applied on the wood surface exhibited the highest antifungal activity [113]. In turn, Scots pine (*P. sylvestris*) and beech (*F. orientalis*) wood specimens impregnated with leaf extracts of Sicilian sumac (*Rhus coriaria* L.), valonia oak (*Quercus macrolepis* L.), and Turkish pine bark (*Pinus brutia* Ten.) showed increased resistance to *T. versicolor* (beech wood) and *G. trabeum* (pine wood) when compared to the untreated control specimens [114]. The antifungal activity of the ethanolic extract of konjac (*Amorphophallus konjac K. Koch*) flying powder, which is a by-product produced during the mechanical processing of konjac flour, was determined against wood decay fungi by Bi et al. [115]. The ethanolic extract of konjac flying powder showed high efficacy against *G. trabeum* and *T. versicolor* on artificial media. Salicylic acid, 2,4,6-trichlorophenol, vanillin, and cinnamaldehyde have been identified in the most active fraction of the extract [115]. The antifungal resistance of wood species treated with various plant extracts is presented in Table 4. 

An interesting group of chemical compounds with antimicrobial activity are phenolic compounds including flavonoids, aromatic acids, and their esters [125,126]. Phenolic compounds are large groups of chemical compounds found in plants that exhibit health benefits and possess wide biological properties including antioxidant, antimicrobial, anticancer, and anti-inflammatory [127,128,129]. Moreover, the literature reports indicate that the regular intake of phenolic compounds may reduce the risk of diabetes and prevent several diseases and physiological syndromes such as cardiovascular and neurogenerative diseases [126,127,129]. The antimicrobial activity of phenolic compounds may also be useful in the development of ecological wood preservatives. Flavonoids—galangin and pinocembrin—exhibited comparable antifungal activity against *G. applanatum*, *L. elegans*, *P. sanguineus*, *S. commune*, and *A. niger* to ketoconazole [93]. Pinocembrin also exhibited strong antifungal activity against *Penicillium italicum* in a dose-dependent manner [130]. Salas et al. [131] examined the antifungal potential of flavonoids isolated from *Citrus* species such as hesperidin, naringenin, and neohesperidin as well as their enzymatically-modified derivatives. The results indicate that the tested phenolic compounds showed diversified activity against *A. flavus* or *Penicillium expansum*, but taking into account that they were obtained as by-products from residues of the citrus industry, they may be interesting antifungal agents [131]. The results of the antifungal activity of chemical compounds isolated from various plant sources are presented in Table 5. 

The activity against pathogenic fungi including mold and wood decay fungi was also demonstrated by other natural compounds of plant origin including coumarins, tar oil from macadamia nut shells, or spirulina. Montagner et al. [139] examined the antifungal activity against *A. fumigatus* and *Fusarium solani* of forty coumarins, and the results indicated that osthenol was characterized by the most effective antifungal potential, which can be associated with the presence of an alkyl group at the C-8 position. Methanolic extract from spirulina (a blue-green algae) inhibited the growth of molds including *A. niger*, *A. flavus*, and *A. fumigatus* [140,141]. Pine wood treated with tar oil obtained from a commercial pyrolysis of macadamia nut shells showed a good protective effect against *T. palustris*, *L. lepideus*, *T. versicolor*, and *P. ostreatus*, with lower values of mass loss than the untreated wood [142]. The chili juice and the extract of habanero chili oleoresins exhibited moderate activity against two sapstain fungi—*S. sapinea* and *L. procerum* [143].

## 3. Animal-Derived Antifungal Agents

Several compounds of animal origin have been reported as potential wood preservatives, mostly in combination with other components. Among the animal-derived compounds, beeswax, animal proteins, or chitosan were used in research on ecological wood protection [21,144,145]. The impregnation of poplar, pine, beach, and lime wood with honeybee wax resulted in a significant reduction in the weight loss of the samples exposed to *T. versicolor* and *N. lepideus* compared to the unprotected control wood samples [144].

An interesting group of animal-derived components are antimicrobial proteins (AMPs), which are small proteins produced by organisms throughout all kingdoms comprising prokaryotes, lower and higher eukaryotes with antifungal, antibacterial, and antiviruses properties [146,147]. Gallerimycin, isolated from the greater wax moth larvae *Galleria mellonella,* exhibited activity against *A. niger*, while ant-derived actinobacterial isolates showed an inhibitory capability against *R. solani* or *Alternaria solani* [146,148]. Hoda et al. [149] reported the activity of extracts (methanolic, acetic acid, and acetone) from the snail *Helix aspersa* against *A. flavus* and *Aspergillus brasiliensis* in a concentration-dependent manner. Chromatographic analysis of these extracts showed that they contained phenolic compounds with antimicrobial properties including hesperidin, genistein, and luteolin, which are characterized by a wide antimicrobial potential [149,150,151,152]. The research described by Ulagesan and Kim [20] indicated that of the seven different snail proteins, the snail *Cryptozona bistrialis* proteins exhibited the most effective activity against *A. fumigatus* and *P. chrysogenum*.

Chitosan is the most frequently studied polymer of animal origin as a component of wood preservatives. The literature data report that the antifungal activity of chitosan as a wood preservative depends on its concentration, molecular weight, the solvent used for the chitosan dissolved, or fungal strain [21,90,153,154,155,156]. The increase in chitosan concentration and molecular weight increases its fungicidal activity [21,154]. The results of research performed by Casado-Sanz et al. [96] indicated that wood treated with chitosan oligomers even at low concentration showed a higher resistance of treated wood against *T. versicolor* compared to the unprotected wood samples, however, the antifungal efficacy of chitosan treatment decreased with the time of exposure to fungus. The activity of medium molecular weight chitosan and chitosan oligomers against *T. versicolor* evaluated in an in vitro growth inhibition experiment showed that chitosan oligomers presented a stronger inhibitory effect, even at a lower concentration than the chitosan with a medium molecular weight [90]. In turn, poplar wood impregnated with chitosan oligomers showed slower degradation caused by *T. versicolor* than wood treated with a medium molecular weight chitosan [90]. Among the three different chitosan samples, two chitosan dissolved in weak acid solutions showed strong antifungal activity against *A. niger* and *Penicillium decumbens*, while chitosan in the form of an aqueous oligomer solution exhibited a 100% reduction in the colony size only against *P. decumbens* in the radial growth inhibitor assay. On the other hand, rubberwood (*Hevea brasiliensis*) treated with an acid chitosan solution showed resistance against *A. niger*, and no resistance against *P. decumbens* [153]. Wood samples impregnated with 5% chitosan solution showed resistance against the brown rot fungi *C. puteana*, *T. versicolor*, and *P. placenta*, while wood treated with a lower concentration of chitosan exhibited no resistance to the tested fungi [21,157].

Sivrikaya et al. [158] examined the potential application of beef tallow, which is a by-product of the rendering of fats in meat production, in wood protection. The authors indicated that the treatment of pine wood with 100% tallow caused an improvement in wood resistance against *C. puteana*, where mass loss for the impregnated wood was 14.24% and unprotected wood was 28.04%. In turn, weight losses for the untreated beech wood and beech block treated with 100% tallow exposed to *T. versicolor* were 30.94 and 16.55%, respectively [158].

## 4. Synthetic Antifungal Agents

The research on ecological wood protection focuses not only on the use of natural compounds, but also on the application of synthetic compounds, but with a low impact on the natural environment. This section discusses the results of studies on the antifungal activity of synthetic compounds for wood protection presented in the literature. The section focuses on three main groups of synthetic agents: silicon compounds, ionic liquids, nanoparticles and nano-compounds, which have been tested in wood protection [7,159,160,161].

### 4.1. Silicon Compounds

Silicon compounds are synthetic molecules, the use of which in wood protection results in the improvement in many wood properties such as dimension stability, the reduction in hydrophilic properties, or increased in fire and fungal resistance [162,163,164,165,166]. De Vetter et al. [167] examined the resistance of pine and beech wood impregnated with six organosilicons against the wood decay fungi—*C. puteana* or *P. placenta* for pine wood and *C. puteana* or *T. versicolor* for beech wood. The results indicated that the higher weight percentage gain of the organosilicon caused increased treated wood resistance to fungi, and the most promising antifungal products were a solvent-based mixture of methyltrimethoxysilane and octyltriethoxysilane and a water-based micro-emulsion of polydimethylsiloxane and triethoxysilane [167]. Pine wood treated with phenyltriethoxysilane showed improved resistance against *P. placenta* compared to the unprotected wood samples [168]. Norway spruce wood samples coated with octadecyltrichlorosilane showed significantly lower mass loss after exposure to *C. puteana* than the uncoated wood. Moreover, the research indicated a linear correlation between the time of wood treatment (30 min, 1, and 2 h) and the antifungal protection of the silicon compound [169]. The results described by Panov and Terziev [168] showed that diethyldiethoxysilane in the form of silanol showed a better result for the durability of pine wood against *P. placenta* than that of wood impregnated with silane diluted with the water-ethanolic solution. In turn, Pries et al. [170] examined the effect of silicones with different functional groups (diamino, carboxyl, carbonyl, betain, and epoxy) on the resistance of pine and beech wood against the decay fungi—*C. puteana* and *T. versicolor*. The results indicated that both the chain length and the functionality of the silicon compounds influenced the fungus durability of the treated wood: the fungus resistance of the treated wood increased with the shorter chain length of silanes, and the carboxy groups imparted a somewhat higher decay resistance than the epoxy and amino groups [170]. The research by Reinprecht and Grznarik [171] showed that pine wood treated with organosilanes—methyltrimethoxysilane, vinyltrimethoxysilane, and propyltrimethoxysilane—exhibited only a slight activity against the mold (*P. brevicompactum*) and decay fungi (*T. versicolor* and *C. puteana*), while wood impregnated with aminopropyltrimethoxysilane showed a significantly higher anti-decay and anti-mold effect. The literature data confirmed that wood treated with aminosilanes showed resistance to decay and mold fungi, and the antifungal activity of these compounds is related, among others, to the presence of an amino group in their molecules [172,173,174].

### 4.2. Ionic Liquids

Ionic liquids (ILs) are remarkable chemical compounds with many applications in various fields of modern science [175]. ILs also possess antimicrobial activity, which has been used in research into the application of ILs as antifungal agents in wood protection. According to the literature reports, various types of ionic liquids have shown activity against wood decay fungi as well as molds, and their antifungal effectivity was related to both the cations and anions of the salt molecule [176,177,178,179]. The activity of 1-butyl-3-methylimidazolium acetate against fungi (*A. versicolor*, *Ch. globosum*, *P. chrysogenum*, and *Penicillium glabrum*) was more effective than 1-butyl-3-methylimidazolium chloride [180]. The results presented in the work of Zabielska-Matejuk et al. [181] indicated that the antifungal properties of ammonium- and triazolium-based ionic liquids depended on their cation and anion structure, and ammonium ILs with a nitrite anion exhibited a stronger fungistatic effect than ammonium nitrates. Imidazolium chlorides (l-alkyl-3-benzyloxymethylimidazolium, 3-alkoxymethyl-1-benzylimidazolium, and 1-alkyl-3-(3-phcnyl-propoxymethyl)imidazolium chlorides) presented activity against *C. puteana*, *T. versicolor*, and *Ch. globosum* in the agar dilution test, and their antifungal action depended on the chain length and their hydrophobicity [177]. In turn, imidazolium compounds with alkoxymethyl and cycloalkoxymethyl substituents and various anions (formats, acetates, propionates) showed activity against *C. puteana*, *T. versicolor*, *Ch. globosum*, *A. niger*, and *S. pythiophila*, and their antifungal action was related to the alkyl chain and the number of carbons in the cycloalkyl ring in IL molecules [178]. Pyridinium-based ion liquids investigated by Stasiewicz et al. [182] exhibited activity against *C. puteana*, *T. versicolor*, and *S. pythiophila*, and their activity against the tested fungi was connected with the position of the substituents in the pyridinium ring. Eight imidazolium chlorides with various alkyl substituents (from methyl to dodecyl) and with a natural component menthol showed diverse activity against wood decay fungi (*C. puteana* and *T. versicolor*) and blue strain fungi (*S. pythiophila*), which depended on the chemical structure of the functional groups [179,183].

### 4.3. Nanosized Particles, Compound, and Substances

In recent years, nanotechnology has also been one of the most extensively developed fields of research, even in wood protection [23,184,185,186]. So far, wood protection has been undertaken using various nanoparticles in the form of single elements such as silver (Ag), copper (Cu), zinc (Zn), boron (B), or in form of chemical compounds including zinc oxide (ZnO), copper oxide (CuO), and titanium dioxide (TiO_2_) [23,90,187].

Pine wood impregnated with silver nanoparticles showed lower values of weight loss caused by *T. versicolor* than the control wood [96]. Silver nanoparticles dispersed in water exhibited a strong protection efficiency of the poplar wood surface against the molds *A. niger*, *P. citrinum*, and *T. viride* [188]. Impregnation with titanium dioxide nanoparticles of eight wood species (*Pinus sylvestris* L., *Abies alba* M., *Juglans regia* L., *Castanea sativa* M., *Prunus avium* L., *Quercus petraea* L., *Fagus sylvatica* L., and *Fraxinus excelsior* L.) prevented the growth of the rot fungi *H. lixii* (white rot) and *M. circinelloides* (brown rot), irrespective of the wood species [189]. Particles of nano-ZnO partially inhibited the growth of fungi (mixture of *A. alternata*, *A. niger*, *P. brevicompactum*, and *Ch. globosum*) on the surface of treated lime tree and maple wood [190]. The treatment of pine wood with an aqueous solution of ZnO nanoparticles at 2.5% and 5.0% concentrations led to an improvement in the resistance to the white rot fungus *G. applanatum* compared to the untreated wood samples [186]. In turn, the impregnation of Scot pine with nano-sized zinc oxide inhibited wood degradation caused by *S. lacrymans* [191]. Additionally, wood impregnated with the nano silver–copper alloy characterized by a higher resistance against mold compared to the unprotected wood samples [192]. Terzi et al. [193] examined the resistance of pine wood treated with various nanoparticles including ZnO, B_2_O_3_, CuO, CeO_2_, TiO_2_, and SnO_2_ against decay (*T. versicolor* and *G. trabeum*) and mold (*A. niger*, *T. harzianum* and *Penicillium pinophilum*) fungi. Mold growth on the wood surface was significantly inhibited by treatment with nano-B_2_O_3_ and nano-ZnO, while treatment with nano-SiO_2_ inhibited *T. harzianum* growth, and that impregnated with nano-CuO and nano-B_2_O_3_ reduced the growth of *T. versicolor*. Moreover, wood treated with all nanoparticles exhibited a lower mass loss caused by the attack of *G. trabeum* compared to the unprotected wood [193]. Rubberwood (*Hevea brasiliensis* Muell Arg.) impregnated with copper oxide and zinc oxide nanoparticles dispersed in propylene glycol effectively inhibited white rot (*T. hirsuta*) and brown rot (*P. meliae*) decay fungi [194]. Pine wood treated with an autoxidized soybean oil polymer containing Ag nanoparticles demonstrated higher decay resistance against *C. puteana* than the unprotected wood [195]. The synthesized garlic-templated fluorescent nanoparticles, prepared through the nano-modification of the garlic extract showed a noticeable antifungal action by inhibiting the growth of the wood decay fungus *T. versicolor*. Moreover, nano-modification enhanced the antifungal activity of garlic extract and removed its odor [196].

## 5. Complex Formulation as Antifungal Agents

The protection of wood with products of plant or animal origin is often associated with important disadvantages, namely, the susceptibility of these components to leaching from the structure of impregnated wood. Therefore, numerous methods have been used to improve their leaching resistance including the addition of hydrophobic or crosslinking agents, thermal modification, micro-capsulation, or the enzyme-catalyzed method [25,197,198]. This section presents examples of formulations whose ingredients can be classified as ecological agents with low impact on human health and the natural environment. The examples of various preparations consisting of both natural substances and synthetic compounds described as ecological agents are presented in Table 6.

## 6. Conclusions

Legal restrictions and the growing awareness of customers regarding the harmfulness to the environment of using wood preservatives registered as pesticides contribute to the search for new preparations and technologies that are more environmentally friendly. Nowadays, there is a growing emphasis on ecological wood protection by using natural substances such as essential oils, plant extracts, plant secondary metabolites, or animal-derivatives such as chitosan. The antifungal activity of natural substances, especially against species of fungi that can attack wood, makes them a more sustainable alternative to protect wood than the toxic biocides that are currently used. However, the fixation of natural substances into the wood structure is limited; therefore, in a wet environment, they may be leached from the wood structure. The susceptibility of natural substances to leaching from impregnated wood is the factor limiting their application in protecting wood used in external conditions. Therefore, in order to limit the leaching of the natural preservatives from treated wood, natural substances are combined with other agents, often of synthetic origin, to limit their leaching. Ecological wood protection products also include silicon compounds, ionic liquids and particles, chemical compounds, and substances with a nanometric size. The production of some synthetic compounds results in toxic by-products. One example may be the production of nanoparticles, the production of which is often associated with the production of harmful by-products. However, more and more often, new ecological methods of obtaining metals with nanometric dimensions are described in the literature. Similarly to natural substances, synthetic compounds can also be leached from wood structure, which is their serious disadvantage in the case of wood used in outdoor conditions.

Currently, research on the acquisition of new, environmentally friendly means of wood protection is extremely important from the point of view of environmental protection. Plants including waste from the agri-food industry can be a rich source of new antimicrobial agents, which would be in line with the adopted zero waste concept. However, an important factor in the development of new ecological wood preservatives, both of natural and synthetic origin, is understanding the mechanism of their antimicrobial activity as well as their environmental fate and toxicity. Therefore, it is mandatory to quantify the rate of accumulation and the release of these agents in the environment.

The presented review shows the data from the literature reports describing the current directions of research on antifungal substances and chemical compounds for ecological wood protection. The paper, of course, does not exhaust the topic of ecological wood preservatives, but the data presented in it can be used to select appropriate measures and develop new, comprehensive preparations for environmentally friendly wood protection.

## Figures and Tables

**Table 1:** A summary of the research on the antifungal activity of essential oils.

Essential Oil	Tested Fungal Strain	Results	References
Ajowan (*Carum copticum*)	Wood decay fungi (*G. trabeum*, *P. placenta*, *T. versicolor*, *C. puteana*, *Ch. globosum*); mold and blue stain fungi (*A. niger*, *A. flavus*, *P. variotii*, *P. chrysogenum*, *A. pullulans*, *T. viride*, *P. commune*)	Agar diffusion plate method indicated that ajowan oil possessed a remarkable activity against mold fungi at the concentration of 0.5%. Beech and pine wood treated with ajowan oil showed resistance against decay fungi and low effect of inhibiting the growth of molds on the wood surfaces.	[30,54,63,64]
Bergamot (*Citrus x limon*)	Wood decay fungi (*C. puteana*, *T. versicolor*, *Ch. globosum*); mold fungi (*A. niger*, *P. commune*)	Beech and pine wood treated with bergamot oil characterized by no growth on wood surface after 10 weeks exposition to *P. commune*. In turn, treated wood showed no resistance against *A. niger* and fungi causing brown and white wood decay.	[30]
Birch (*Betula pendula*)	Wood decay fungi (*C. puteana*, *T. versicolor*); mold fungi (*A. niger*, *P. brevicompactum*)	The effective concentration of birch oil against growth of *C. puteana* was 3.5%, while against *T. versicolor*, *A. niger* and *P. brevicompactum* was 10% on treated filter paper. The mass loss of beech wood treated with 10% birch oil caused by *C. puteana* was 0.08%, compared to the control wood—27.01%.	[16]
Cedar wood (*Juniperus mexicana*)	Wood decay fungi (*C. puteana*, *T. versicolor*, *Ch. globosum*); mold fungi (*A. niger*, *P. commune*)	Beech and pine wood treated with cedar wood oil did not show resistance against both decay and mold fungi.	[30]
Clove (*Eugenia caryophyllata*, *Syzygium aromaticum)*	Wood decay fungi (*G. trabeum*, *T. versicolor*, *C. puteana*, *T. hirsuta*, *L. sulphureus*); mold fungi (*A. niger. P. brevicompactum*, *A. flavus*, *A. fumigatus*, *P. variotii*, *T. viride*)	Clove oil at 0.5% concentration was effective against molds, causing growth inhibition in the range from 60% (*T. viride*) to 84% (*A. niger*) tested by the agar diffusion plate method. Clove oil at a 400 µg/mL concentration totally inhibited the growth of *G. trabeum* and *T. versicolor* tested by the diffusion method. The MIC of clove oil against *A. flavus* was 0.64 µg/mL, while against *A. fumigatus* and *A. niger*, it was 0.32 µg/mL. The effective concentration of clove oil against the growth of *C. puteana*, *A. niger*, and *P. brevicompactum* was 3.5%, while against *T. versicolor*, it was 10% on treated filter paper. The mass loss of beech wood treated with 10% clove oil caused by *C. puteana* was 0.04% compared to the control wood—27.01%.	[16,57,60,64,65]
Dill (*Anethum graveolens*)	Wood decay fungi (*G. trabeum*, *P. placenta*, *T. versicolor*); mold and blue stain fungi (*A. niger*, *P. chrysogenum*, *A. pullulans*, *T. viride*)	Southern yellow pine treated with dill oil showed resistance against tested decay, mold, and blue stain fungi.	[54,63]
Eucalyptus (*Eucalyptus camaldulensis*, *E. globulus*)	Wood decay fungi (*C. puteana*, *T. versicolor*, *A. alternata*, *Ch. globosum*); mold fungi (*F. subglutinans*, *A. niger*, *T. viride*, *P. commune*)	Beech and pine wood treated with eucalyptus oil showed resistance against *Ch. globosum* and *P. commune*, and no resistance against *C. puteana*, *T. versicolor*, and *A. niger*. The wood (*P. sylvestris*, *P. rigida*, and *F. sylvatica*) treated with eucalyptus oil showed better resistance against *Ch. globosum* and *A. niger* than against *T. viride*.	[30,51]
Geranium (*Pelargonium graveolens*)	Wood decay fungi (*C. puteana*, *C. versicolor*, *P. placenta*, *G. trabeum*, *T. hirsuta*, *L. sulphureus*, *T. versicolor*, *Ch. globosum*); mold and blue stain fungi (*A. niger*, *P. chrysogenum*, *A. pullulans*, *T. viride*, *P. commune*)	Beech and pine wood treated with geranium oil showed resistance against decay fungi—*C. puteana*, *T. versicolor*, *Ch. globosum* (except of treated beech against *C. puteana*), and *P. commune*. Southern yellow pine treated with 100% geranium oil showed a resistance against decay fungi (*G. trabeum*, *P. placenta* and *T. versicolor*) with no weight loss in the wood specimens. Pine wood treated with geranium oil exhibited resistance against mold fungi (*P. chrysogenum*, *A. niger* and *T. viride*).	[30,49,54,57,63]
Lavender (*Lavandula angustifolia*)	Wood decay fungi (*C. puteana*, *T. versicolor*, *Ch. globosum*); mold fungi (*A. niger*, *P. brevicompactum*, *P. commune*)	The effective concentration of lavender oil against the growth of *C. puteana* was 10%, while against *T. versicolor* and *A. niger*, it was 100% on the treated filter paper. Beech and pine wood treated with lavender oil showed resistance against decay fungi (*C. puteana*, *T. versicolor*, *Ch. globosum*) and molds (*A. niger*, *P. commune*). The mass loss of beech wood treated with 10% lavender oil caused by *C. puteana* was 8.02% compared to the control wood—27.01%.	[16,30]
Lemongrass (*Cymbopogon flexuosus*, *C. winterianus*)	Wood decay fungi (*G. trabeum*, *P. placenta*, *T. versicolor*, *C. puteana*, *Ch. globosum*); mold and blue stain fungi (*A. niger*, *P. chrysogenum*, *A. pullulans*, *T. viride*, *P. commune*)	Beech and pine wood treated with lemongrass oil showed resistance against decay fungi (*C. puteana*, *T. versicolor*, *Ch. globosum*) and molds (*A. niger*, *P. commune*). Pine wood treated with 10% and 100% lemongrass oil showed resistance against decay fungi—*G. trabeum*, *P. placenta*, and *T. versicolor*. In turn, the inhibitory effect on the surface of the wood specimens treated with lemongrass oil against molds (*A. niger*, *T. viride* and *P. chrysogenum*) was low.	[30,54,63]
Neem (*Azadirachta indica*)	Mold fungi (*A. niger*, *A. flavus*, *P. variotii*, *T. viride*)	Neem oil at a 0.5% concentration possessed a remarkable antifungal activity against all of the tested fungi, which was evaluated for its ability to inhibit weight loss by soil wood block tests.	[64]
Oregano (*Origanum vulgare*)	Wood decay fungi (*T. hirsuta*, *L. sulphureus*, *C. puteana*, *T. versicolor*); mold fungi (*A. niger*, *P. brevicompactum*)	The effective concentration of oregano oil against growth of *T. versicolor*, *A. niger* and *P. brevicompactum* was 10%, while against *C. puteana*—1%, on treated filter paper. Oregano oil at 400 µg/mL concentration showed 100% inhibition of *T. hirsuta* and *L*. *sulphureus* growth. The mass loss of beech wood treated with 10% oregano oil caused by *C. puteana* was 0.02%, compared to control wood—27.01%.	[16,57]
Peppermint (*Mentha* x *piperita*)	Wood decay fungi (*T. versicolor*, *C. puteana*, *Ch. globosum*) and mold fungi (*A. niger*, *P. chrysogenum*, *P. commune*)	Beech and pine wood treated with peppermint oil showed resistance against *P. commune* and moderate activity against *A. niger*. Treated beech exhibited activity against *C. puteana* and no resistance against *T. versicolor*, while impregnated pine wood showed resistance against *T. versicolor* and moderate resistance against *C. puteana*.	[30,62]
Pinus (*Pinus rigida*—wood)	Wood decay fungi (*A. alternata*, *Ch. globosum*); mold fungi (*F. subglutinans*, *A. niger*, *T. viride*)	The wood (*P. sylvestris*, *P. rigida* and *F. sylvatica*) treated with Pinus oil showed resistance against all tested fungi.	[51]
Rosemary (*Rosmarinus officinalis*)	Wood decay fungi (*G. trabeum*, *P. placenta*, *T. versicolor*); mold and blue stain fungi (*A. niger*, *A. flavus*, *A. ochraceus*, *P. chrysogenum*, *A. pullulans*, *T. viride*)	The inhibitory effect on the surface of the wood specimens treated with rosemary oil against molds (*A. niger*, *T. viride* and *P. chrysogenum*) was low. Pine wood treated with 10% and 100% rosemary oil showed resistance against decay fungi—*G. trabeum*, *P. placenta*, and *T. versicolor*, with no weight loss of the wood samples.	[54,63,66]
Sage (*Salvia officinalis*)	Wood decay fungi (*C. puteana*, *T. versicolor*); mold fungi (*A. niger*, *P. brevicompactum*)	The effective concentration of sage oil against the growth of decay fungi was 100%, while against molds, sage oil was non-active on the treated filter paper. The mass loss of beech wood treated with 10% sage oil caused by *C. puteana* was 17.80% compared to the control wood—27.01%.	[16]
Savory (*Satureja hortensis*)	Wood decay fungi (*C. puteana*, *T. versicolor*); mold fungi (*A. niger*, *P. brevicompactum*)	The effective concentration of savory oil against the growth of *C. puteana* was 3.5%, while against *T. versicolor* and molds, it was 10% on the treated filter paper. The mass loss of beech wood treated with 10% savory oil caused by *C. puteana* was 0.07% compared to the control wood—27.01%.	[16]
Sweet flag (*Acorus calamus*)	Wood decay fungi (*C. puteana*, *T. versicolor*); mold fungi (*A. niger*, *P. brevicompactum*)	The effective concentration of sweet flag oil against the growth of *C. puteana* and *P. brevicompactum* was 3.5%, while against *T. versicolor* and *A. niger*, it was 10% on the treated filter paper. The mass loss of beech wood treated with 10% sweet flag oil caused by *C. puteana* was 0.04% compared to the control wood—27.01%.	[16]
Tea tree (*Melaleuca alternifolia*)	Wood decay fungi (*G. trabeum*, *P. placenta*, *T. versicolor*, *C. puteana*, *Ch. globosum*); mold and blue stain fungi (*A. niger*, *P. chrysogenum*, *A. pullulans*, *T. viride*, *P. brevicompactum*, *P. commune*)	The inhibitory effect on the surface of wood specimens treated with tea tree oil against molds (*A. niger*, *T. viride,* and *P. chrysogenum*) was low. The effective concentration of tea tree oil against the growth of decay (*C. puteana* and *T. versicolor*) and mold fungi (*A. niger* and *P. brevicompactum*) was 100%. The mass loss of beech wood treated with 10% tea tree oil caused by *C. puteana* was 8.52% compared to the control wood—27.01%.	[16,30,54,63]
Thyme (*Thymus vulgaris*)	Wood decay fungi (*T. hirsuta*, *L. sulphureus*, *G. trabeum*, *P. placenta*, *T. versicolor*, *C. puteana*, *Ch. globosum*); mold fungi (*A. niger*, *P. chrysogenum*, *A. pullulans*, *T. viride*, *P. brevicompactum*, *P. commune*)	The effective concentration of thyme oil against the growth of *C. puteana* and *P*. *brevicompactum* was 3.5%, while against *T. versicolor* and *A. niger* it was 10%. The mass loss of beech wood treated with 10% thyme oil caused by *C. puteana* was 0.06% compared to the control wood—27.01%. Pine wood treated with 10% and 100% thyme oil showed resistance against decay fungi—*G. trabeum*, *P. placenta*, and *T. versicolor*, with no weight loss of the wood samples.	[16,30,54,57,63]

**Table 2:** A summary of the research on the antifungal activity of essential oil components.

Chemical Compounds	Tested Fungal Strain	Results	References
α-pinene	Wood decay fungi (*T. hirsuta*, *S. commune*, *P. sanguineus*); mold fungi *(A. niger*, *A. flavus*, *A. ochraceus*)	The diameter of the inhibition zone of *A. flavus* was in the range from 0.85 cm (2.5 µL concentration) to 3.61 cm (20 µL concentration). Pinene at a 50 µg/mL concentration did not show activity against wood decay fungi in the fluid medium test.	[17,66]
Borneol	Wood decay fungi (*T. hirsuta*, *S. commune*, *P. sanguineus*)	Borneol at 50 µg/mL concentration did not show activity against wood decay fungi in the fluid medium test.	[17]
Carvacrol	Wood decay fungi (*T. versicolor*, *C. puteana*, *T. hirsuta*, *S. commune*, *P. sanguineus*, *L. sulphureus*, *A. alternata*); mold fungi (*A. flavus*, *A. fumigatus*, *A. niger*, *A. ochraceus*, *P. citrinum*, *P. chrysogenum*, *F. oxysporum*)	IC_50_ values of carvacrol against *S. commune* and *P. sanguineus* were 53.6 and 71.7 µg/mL, respectively. In turn, IC_50_ values of carvacrol against *T. hirsuta* and *L. sulphureus* were 33.6 and 17.2 μg/mL, respectively. The MIC of carvacrol against *T. versicolor* and *C. puteana* was 1.25 and 0.625 mmol/L, respectively. Carvacrol showed activity against *Aspergillus* species with MIC vales from 50 (*A. niger*) to 100 µg/mL (*A. fumigatus*, *A. flavus*, *A. ochraceus*).	[17,57,67,69,71]
Cinnamaldehyde	Wood decay fungi (*L. betulina*, *L. sulphureus*, *C. versicolor*, *P. coccineus*, *T. abietinum*, *O. lowei*, *A. taxa*, *F. pinicola*, *P. schweinitzii*, *T. hirsute*, *T. palustris*, *T. versicolor*, *G. trabeum*); mold fungus (*A. niger*)	IC_50_ value of cinnamaldehyde against *L. betulina* was 0.65 mM and against *L. sulphureus* was 0.23 mM. Cinnamaldehyde at 100 ppm concentration showed high activity against wood rot fungi including *C. puteana*, *T. versicolor*, *P. coccineus*, *A. taxa*, and *P. schweinitzii*. Cinnamaldehyde below a 100 µg/mL concentration totally inhibited the growth of *T. hirsute* and *L. sulphureus*. The MIC of cinnamaldehyde against *T. versicolor* and *C. puteana* was 1.25 and 0.625 mmol/L, respectively.	[52,53,57,72,73,74]
Citral	Wood decay fungi (*T. hirsuta*, *S. commune*, *P. sanguineus*, *L. sulphureus*); molds (*T. viride*, *A. niger*, *P. citrinum*)	The MIC of citral against *P. citrinum*, *T. viride*, and *A. niger* resulting in the inhibition of fungal growth on the bamboo wood surfaced was 0.180, 0.265, and 0.226 mg/mL, respectively. IC_50_ values of citral against *T. hirsuta*, *S. commune*, and *P. sanguineus* were 184.5, 207.5, and 178.5 µg/mL, respectively.	[17,57,70]
Citronellol	Wood decay fungi (*P. placenta*, *G. trabeum*, *T. hirsuta*, *S. commune*, *P. sanguineus*, *L. sulphureus*); mold fungi (*A. niger*, *P. chrysogenum*, *T. viride*)	IC_50_ values of citronellol against *T. hirsuta* and *L. sulphureus* were 80.5 and 61.6 µg/mL, respectively. IC_50_ value of monoterpene against decay wood fungi was in a range from 190.2 µg/mL (*P. sanguineus*) to 219.5 µg/mL (*T. hirsuta*).	[17,50,57]
Eugenol	Wood decay fungi (*T. versicolor*, *C. puteana*, *T. hirsuta*, *S. commune*, *P. sanguineus*, *L. betulina*, *L. sulphureus*, *C. versicolor*, *A. alternata*); mold fungi (*A. niger*, *A. flavus*, *A. ochraceus*, *A. fumigatus*, *P. citrinum*, *P. chrysogenum*, *F. oxysporum*)	IC_50_ values of eugenol against *T. hirsuta*, *S. commune*, and *P. sanguineus* were 85.1, 122.2, and 137.7 µg/mL, respectively. Eugenol at the concentration of 100 µg/mL showed high activity against *L. betulina* and *L. sulphureus*. IC_50_ value of eugenol against *L. betulina* was 0.37 mM and against *L. sulphureus* was 0.25 mM. The MIC value of eugenol against *T. versicolor* and *C. puteana* was 5.0 and 1.25 mmol/L, respectively.	[17,52,53,57,66,67,68,69,71]
Geraniol	Wood decay fungi (*P. placenta*, *G. trabeum*, *T. hirsuta*, *S. commune*, *P. sanguineus*, *L. sulphureus*); mold fungi (*A. niger*, *P. chrysogenum*, *T. viride*)	IC_50_ values of geraniol against *T. hirsuta*, *S. commune*, and *P. sanguineus* were 189.3, 224.4, and 367.6 µg/mL, respectively. IC_50_ values of geraniol against *A. niger* was 128.7 mg/L.	[17,50,57,75]
Menthol	Wood decay fungi (*T. hirsuta*, *S. commune*, *P. sanguineus*, *T. versicolor*, *A. alternata*); mold fungi (*A. niger*, *P. chrysogenum*, *A. fumigatus*, *A. flavus*, *P. citrinum*, *F. oxysporum*, *A. ochraceus*)	Menthol did not show activity against decay wood fungi—*T. hirsuta*, *S. commune*, and *P. sanguineus*. The MIC of menthol against *A. niger* and *T. versicolor* was 250 µL/mL, while against *P. chrysogenum* was 150 µL/mL. The MIC value of menthol against *A. flavus* and *A. ochraceus* was 100 µg/mL, 150 µg/mL against *A. fumigatus*, and 450 µg/mL against *A. alternata*.	[17,62,67]
Nerol	Wood decay fungi (*T. hirsuta*, *S. commune*, *P. sanguineus*); mold fungi (*A. flavus*)	Nerol in a concentration higher than 7.5 μL totally inhibited the growth of the *A. flavus* strain and the diameter of inhibition zone was in the range from 2.68 cm (2.5 µL concentration) to 6.09 cm (20 µL concentration). IC_50_ values of nerol against *T. hirsuta*, *S. commune*, and *P. sanguineus* were 166.3, 337.6, and 242.7 µg/mL, respectively.	[17,75]
Thymol	Wood decay fungi (*P. placenta*, *G. trabeum*, *T. versicolor*, *C. puteana*, *A. alternata*); mold fungi (*A. niger*, *P. chrysogenum*, *T. viride*, *A. flavus*, *A. fumigatus*, *P. citrinum*, *F. oxysporum*)	IC_50_ values of thymol against *S. commune* and *P. sanguineus* were 67.1 and 116.8 µg/mL, respectively. The MIC value of thymol against *T. versicolor* and *C. puteana* was 1.25 and 0.313 mmol/L, respectively. IC_50_ of thymol against *T. hirsuta* and *L. sulphureus* was 45.8 and 23.2 µg/mL, respectively. In turn, IC_50_ values of thymol against *A. niger* was 23.80 mg/L.	[50,57,67,69,71,75,76]
Vanillin	Wood decay fungi (*T. versicolor*, *C. puteana*); mold fungi (*A. flavus*, *A. fumigatus*)	The MIC value of vanillin against *T. versicolor* and *C. puteana* was 10 mmol/L. Inhibitory effect of vanillin on *A. flavus* and *A. fumigatus* at a concentration 1000 µg/mL was 46.34 and 100%, respectively.	[69,71]

**Table 3:** A summary of the research on the antifungal activity of plant extracts.

Plant Source	Tested Fungal Strain	Results	References
*Eriosema robustum*	*A. fumigatus*	The MIC of crude ethanolic extract against *A. fumigatus* was 0.63 mg/mL, while the MIC for the eight compounds identified in this extract was in a range from 65 (6-prenylpinocembrin) to 250 µg/mL (orostachyscerebroside A).	[105]
*Sequoia sempervirens*	*G. trabeum*, *T. versicolor*	Among the extracts prepared using various solvents (water, ethanol, acetone, ethyl acetate, and dichloromethane), the acetone-soluble extract and the ethyl acetate-soluble fraction of the ethanol extract caused the greatest reduction in the growth of both tested fungi.	[106]
*Fagus sylvatica* L.	*G. trabeum*, *T. versicolor*	The paper disc screening test of the methanolic extracts indicated that both wound-wood as well as to healthy sapwood possessed fungicidal potential against the tested fungi. In turn, the extracts of the reaction zones did not exhibit a corresponding inhibitory effect toward the examined fungi.	[107]
*Bagassa guianensis* Aubl (heartwood)	*P. sanguineus*	The ethyl acetate extract showed activity against the wood decay fungus and exhibited a lower MIC value (2 µg/mL) than the six components isolated from this extract including 6-O-methyl-moracin N (8 µg/mL), moracin N (4 µg/mL), and oxyresveratrol (32 µg/mL).	[108]
*Taiwania cryptomerioides* Hayata	*P. noxius*	The hexane-soluble fraction demonstrated a significant inhibition of the growth of wood brown rot fungus among the four examined fractions (hexane, ethyl acetate, butanol, and water) using the agar dilution method. Moreover, constituents of the hexane-soluble fraction (ferruginol, T-cadinol, α-cadinol, and T-muurolol) exhibited excellent antifungal activities against *P. noxius* with IC_50_ values equal 16.9, 25.8, 33.8, and 50.6 µg/mL, respectively.	[109]
*Juniperus virginiana* (heartwood)	*T. versicolor*, *G. trabeum*	Among the four extracts obtained using solvents with different polarity (hexane, chloroform, ethyl acetate, methanol), the hexane and chloroform soluble fraction of the extract showed a high inhibitory effect on the growth of the tested wood decay fungi. The component of extracts (mainly cedrol and thujopsene) exhibited an inhibitory effect against the tested fungi.	[110]
*Dalbergia congestiflora* Pittier (heartwood)	*T. versicolor*	The hexane extract completely inhibited the growth of the tested wood decay fungus, and the main component of this extract was the isoflavonoid—medicarpin.	[111]

**Table 4:** A summary of the research on the antifungal activity of wood treated with plant extracts.

Plant Source	Tested Fungal Strain	Wood Species	Results	References
*Lawsonia inermis*	*G. lucidum*, *S. rolfsii*	*Vitex doniana*, *Triplochiton scleroxylon*	Wood samples treated with extracts, both from the bark and leaves exhibited lower values of weight loss caused by the action of tested fungi in comparison to the untreated wood samples.	[116]
*Acacia dealbata*	*C. puteana*	Scots pine (*P. sylvestris* L.)	The wood samples were impregnated with extracts (methanolic and aqueous) from the bark, sapwood, and heartwood of *A. dealbata* at 3 and 5% concentration. The wood treated with 5% methanolic extracts from bark showed the lowest mass loss (7.45%), while the highest weight loss (23.5%) was determined for wood treated with 3% aqueous extract from sapwood.	[117]
*Robinia pseudoacacia*	*T. versicolor*	European beech (*F. sylvatica* L.)	The treated wood was characterized by higher resistance (mass loss of 12.7%) against the tested fungus compared to the untreated wood samples (mass loss of 43.6%).	[118]
*Eucalyptus camaldulensis* (aerial parts)	*Fusarium culmorum*, *Rhizoctonia solani*, *P. chrysogenum*	*Melia azedarach*	The wood treated with the n-hexane extract showed growth inhibition of the tested fungi. The main components of the extract were β-fenchol, eucalyptol, and subinene.	[119]
*Vitex agnus-castus* (leaves)	*F. culmorum*, *R. solani*, *P. chrysogenum*	*Melia azedarach*	The impregnation of wood with the n-hexane extract showed a higher resistance against the tested fungi compared to the unprotected wood. The main ingredients of the extract were eucalyptol, β-caryophyllene, and β-sitosterol.	[119]
*Juniperus virginiana L.*	*P. placenta*, *G. trabeum*	Southern pine	The wood treated with cedar extract obtained by ethanolic extraction and by liquid carbon dioxide extraction showed a higher resistance against the tested fungi compared to the control wood samples.	[120]
*Cupressus sempervirens*	*T. harzianum*	*Acacia saligna*	The treated wood with the methanolic extract of *C. sempervirens* wood showed an inhibition zone against the growth of *T. harzianum* around the treated wood at the concentrations of 5, 10, and 20%.	[121]
*Usnea filipendula*	*C. puteana*	Scots pine (*P. sylvestris* L.)	The wood samples treated with the methanolic and aqueous extract of lichen were characterized by lower weight loss than the control wood samples.	[122]
*Viscum album*	*C. puteana*	Scots pine (*P. sylvestris* L.)	The wood impregnated with methanolic and aqueous extracts from the leaves of mistletoe showed lower mass loss values compared to the mass loss of the untreated wood.	[122]
*Ocotea lancifolia*	*T. versicolor*, *G. trabeum*	Downy birch (*Betula pubescens*)	Wood veneers impregnated with ethanolic extract from the leaves of the native Brazilian tree and ethyl acetate phenolic-rich fraction of this extract at a concentration of 4% showed higher resistance to wood-destroying fungi compared to the untreated wood veneers.	[123]
*Musa paradisiaca* L.	*F. culmorum*, *R. solani*	*Melia azedarach*	The wood treated with the 3% methanolic extract of *M. paradisiaca* peels showed activity against *F. culmorum* and *R. solani*. The mycelial growth inhibition percentages reached 68.88% for *F. culmorum* and 94.07% for *R. solani*.	[124]

**Table 5:** A summary of the research on the antifungal activity of chemical compounds isolated from plant sources.

Chemical Compound	Plant Source	Tested Fungal Strain	Results	References
Icthyothereol acetate	*Blumea balsamifera*	*A. niger*	The compound possessed moderate activity against *A. niger* with an activity index of 0.4 at a mass of 30 µg determined by the agar cup method.	[132]
4′-methoxy-5,7-dihydroxyflavone 6-C-glucoside	*Aquilegia vulgaris*	*A. niger*	The antimicrobial activity of isocytisoside (4′-methoxy-5,7-dihydroxyflavone 6-C-glucoside) tested by the method of series dilutions against *A. niger* was 62·5 µg/mL.	[133]
(3R)-5,7,2′,3′-tetrahydroxy-4′-methoxy-5′-prenylisoflavanone	*Geoffroea decorticans*	*A. flavus*, *A. parasiticus*, *A. nomius*	The percentage of hyphal radial growth inhibition produced by the compound was 31.2% for *A. flavus*, 40.3% for *A. parasiticus*, and 60.8% for *A. nomius*.	[134]
(3R)-7-2′-3′-trihydroxy-4′-methoxy-5′-prenylisoflavanone	*Geoffroea decorticans*	*A. flavus*, *A. parasiticus*, *A. nomius*	The percentage of hyphal radial growth inhibition caused by the compound was 28.9% for *A. flavus*, 35.8% for *A. parasiticus*, and 57.2% for *A. nomius*.	[134]
Baicalein	*Scutellaria baicalensis* Georgi (root)	*A. fumigatus*	Baicalein showed antifungal activity toward *A. fumigatus* and the minimal inhibitory concentration (MIC_50_) was 0.23 mM.	[135]
Wogonin	*Scutellaria baicalensis* Georgi (root)	*A. fumigatus*	Wogonin exhibited antifungal activity toward *A. fumigatus* and the minimal inhibitory concentrations (MIC_50_) was 0.23 mM.	[135]
Phenolic compounds	*Stenoloma chusanum* (L.) Ching	*A. niger*	The minimum inhibitory concentration (MIC) of 4-*O*-β-d-(6-*O*-gentisoylglucopyranosyl) vanillic acid, vanillic, syringic, and gentisic acids against *A. niger* determined by the test tube dilution method on dextrose agar was 100 µg/mL.	[136]
Phenolic compounds	*Cynara scolymus* L.	*A. niger*	The MIC of chlorogenic acid, cynarin, 3,5-di-*O*-caffeoylquinic acid, and luteolin 7-rutinoside against *A. niger* was 100 µg/mL, the MIC of 4,5-di-*O*-caffeoylquinic acid, apigenin 7-rutinoside, and apigenin-7-*O*-β-d-glucopyranoside was 200 µg/mL, and the MIC of cynaroside was 50 µg/mL.	[137]
Phenolic compounds	*Diospyros virginiana* (fruits)	*A. fumigatus*, *A. versicolor*, *A. ochraceus*, *A. niger*, *P. funiculosum*	The phenolic compounds isolated from the *D. virginana* extract including m-gallate, gallic acid, luteolin, quercetin, myricetin, myricetin 3-*O*-α-rhamnoside, myricetin 3-*O*-β-glucoside, and myricetin 3-*O*-β-glucuronide demonstrated significant activity against molds.	[138]

**Table 6:** A summary of the research on the formulations applied in wood protection.

Preparation	Tested Fungal Strain	Wood Species	Results	References
Caffeine—silicon compounds (AATMOS)	*C. puteana*	Scots pine (*P. sylvestris* L.)	Mass loss of the unleached treated wood exposure to *C. puteana* was 1.6% compared to the control wood—42.3%. After the leaching procedure (EN 84) mass loss of the treated wood was 2.2% and the weight loss of unprotected wood was 46.9%.	[199]
Caffeine—propolis—silicon compounds (MTMOS and OTEOS)	*C. puteana*	Scots pine (*P. sylvestris* L.)	Mass loss of the unleached treated wood was 7.18% compared to the unprotected wood—50.5%, while the weight loss of wood after leaching (EN 84) was 1.61% for the treated wood and 49.7% for the untreated wood.	[26]
Chitosan—cinnamaldehyde	*A. niger*	Poplar (*Populus tomentosa* Carr.)	Wood treated with different molar ratios of cinnamaldehyde and chitosan showed different activity against *A. niger*, which caused the inhibition of fungus growth from 16.7% (0.5:1.0 ratio) to 95.8% (3.0:1.0 ratio).	[200]
Chitosan—propolis—nanoAg	*T. versicolor*	Poplar (*Populus* spp.)	Mass losses of the treated wood were in the range from 0% (after 5 days of fungal exposure) to 39.94% (after 30 days of fungal exposure).	[90]
Genipin—chitosan	*G. trabeum*, *P. placenta*, *T. versicolor*, *I. lacteus*	Southern pine and poplar	Mass loss of the treated wood caused by all fungal species decreased with an increasing concentration of the formulation regardless of the leaching procedure (AWPA E11). However, all treated wood samples recorded significantly lower weight loss compared to the unprotected samples.	[198]
Methyl-β-cyclodextrin—eugenol	*P. placenta*, *G. trabeum*	Southern pine wood	Mass loss of the wood treated with eugenol and 50% solution of MβCD caused by *P. placenta* was 4.84%, and by *G. trabeum*—6.12%.	[24]
Methyl-β-cyclodextrin—cinnamaldehyde	*P. placenta*, *G. trabeum*	Southern pine wood	Mass loss of the wood treated with cinnamaldehyde and 50% solution of MβCD caused by *P. placenta* was 5.50%, and by *G. trabeum*—6.86%.	[24]
Methyl-β-cyclodextrin—carvacrol	*P. placenta*, *G. trabeum*	Southern pine wood	Mass loss of the wood treated with carvacrol and 50% solution of MβCD caused by *P. placenta* was 8.40%, and by *G. trabeum*—7.94%.	[24]
Methyl-β-cyclodextrin—thymol	*P. placenta*, *G. trabeum*	Southern pine wood	Mass loss of the wood treated with thymol and 50% solution of MβCD caused by *P. placenta* was 7.75%, and by *G. trabeum*—6.75%.	[24]
Nano-CuO—extract of *Lantana camara*	*T. hirsuta*, *P. placenta*	Rubberwood (*H. brasiliensis*)	Mass loss of the wood treated with the formulation caused by *T. hirsuta* was 15.01% compared to the untreated wood—28.28%, and the weight loss caused by *P. placenta* was 23.12% for the treated and 51.59% for untreated wood.	[201]
Nano-CuO—extract of *Nerium oleander*	*T. hirsuta*, *P. placenta*	Rubberwood (*H. brasiliensis*)	Mass loss of the wood treated with the formulation caused by *T. hirsuta* was 24.75%, compared to the untreated wood—28.28%, and the weight loss caused by *P. placenta* was 13.19% for the treated and 51.59% for the untreated wood.	[201]
Nano-ZnO—essential oils (clove, oregano, thyme oils)	*Ch. globosum*, *A. niger*, *P. brevicompactum*, *A. alternata*	Lime tree (*T. cordata*), maple (*A. pseudoplatanus* L.)	Clove and oregano oils mixed with ZnO significantly improved the resistance of treated wood against a mixture of tested fungi, mainly in the first days of the test.	[190]
Linear poly(amidoamine)s—nanoAg	*C. puteana*, *T. versicolor*	Scots pine (*P. sylvestris* L.), beech (*F. sylvatica* L.)	The wood treated with the formulation after exposure to the tested fungi showed a weight loss well below 5%.	[202]
Propolis extract—silicon compounds	*C. puteana*	Scots pine (*P. sylvestris* L.)	Wood treated with propolis and VTMOS/MTMOS after exposure to *C. puteana* showed a mass loss of 3.7%, wood treated with propolis and MTMOS/MPTMOS before and after leaching (EN 84) were characterized by a weight loss equal to 3.8 and 3.0%, respectively. Pine wood impregnated with propolis and VTMOS/TEOS showed a mass loss of 3.3% (before leaching) and 3.5% (after leaching—EN 84), while wood impregnated with propolis and MPTMOS/TEOS were characterized by a mass loss equal to 2.9 and 3.2% (EN 84).	[203,204,205]
Salicylic acid—silica microcapsules	*T. versicolor*, *G. trabeum*	Poplar (*P. nigra* L.)	Mass losses of the untreated wood attacked by white rot fungi and brown rot fungi were 42.50% and 62.82%, respectively, while weight losses of the leached treated wood were 14.42% and 15.87% respectively.	[25]
Sorbitol—citric acid	*P. placenta*, *T. versicolor*, *C. puteana*	Scots pine (*P. sylvestris* L.), European beech (*F. sylvatica*)	Treatment of wood with sorbitol and citric acid caused an increase in the decay resistance against brown rot and white rot fungi.	[206,207,208]
Thymol—laccase	*A. niger*	Bamboo (*P. pubescens*)	Impregnation of wood with thymol with laccase improved the resistance to mold compared to the unprotected wood. Moreover, wood treated with thymol with laccase exhibited a higher resistance to mold than wood treated with thymol alone, even after the leaching procedure.	[76]
Vanillin—laccase	*T. versicolor*, *G. trabeum*	Poplar (*P. nigra* L.)	The weight loss of wood exposed to white and brown rot fungi decreased from 46 and 13% to 9 and 4% for the treated wood, respectively.	[209]

## Data Availability

No experimental data are reported in the review manuscript.
